# Outcomes of a clinical diagnostic algorithm for management of ambulatory smear and Xpert MTB/Rif negative HIV infected patients with presumptive pulmonary TB in Uganda: a prospective study

**DOI:** 10.11604/pamj.2016.23.154.7995

**Published:** 2016-03-31

**Authors:** Simon Walusimbi, Fred Semitala, Freddie Bwanga, Melles Haile, Ayesha De Costa, Lucian Davis, Moses Joloba, Sven Hoffner, Moses Kamya

**Affiliations:** 1 Department of Microbiology, Makerere University College of Health Sciences, Kampala, Uganda; 2 Department of Public Health Sciences, Karolinska Institute, Solna, Sweden; 3 Makerere University Joint AIDS Program, Kampala, Uganda; 4 Department of Internal Medicine, Makerere University College of Health Sciences, Kampala, Uganda; 5 Department of Microbiology, Public Health Agency of Sweden, Solna, Sweden; 6 University of California San Francisco, Pulmonary and Critical Care Medicine, San Francisco, United States

**Keywords:** HIV, TB, outcomes, algorithm, strategy, smear-negative, Xpert-negative

## Abstract

**Introduction:**

Diagnostic guidelines for Tuberculosis (TB) in HIV infected patients previously relied on microscopy where the value of initial antibiotic treatment for exclusion of pulmonary TB (PTB) was limited. New guidelines rely on the Xpert MTB Rif test (Xpert). However, the value of the antibiotic treatment remains unclear particularly in individuals who are smear-negative and Xpert-negative-given Xpert has only moderate sensitivity for smear-negative PTB. We assessed an algorithm involving initial treatment with antibiotics prior empiric TB treatment in HIV patients with presumptive PTB who were both smear and Xpert negative.

**Methods:**

We performed a prospective study with six month follow-up to establish patient response to a course of broad spectrum antibiotics prior empiric TB treatment between March 2012 and June 2013. We calculated the proportion of patients who responded to the antibiotic treatment and those who did not. We computed the crude and adjusted odds ratios with their 95% confidence intervals, for response to the antibiotic treatment on various patient characteristics. We report treatment outcomes for patients who received broad spectrum antibiotics only or who were initiated empiric TB treatment.

**Results:**

Our cohort comprised 162 smear-negative and Xpert-negative patients, of whom 59% (96 of 162) were female, 81% (131 of 162) were on antiretroviral therapy (ART) for a median of 8.7 months. Overall, 88% (141 of 160) responded to the antibiotic treatment, 8% (12 of 160) got empiric TB treatment and 4% (7 out of 160) were treated for other respiratory disease. The odds of improvement on antibiotics were lower in patients with advanced HIV disease than in patients with early HIV disease. Adjusted odds ratios were significant for HIV clinical stage (AOR; 0.038,) and duration on ART (AOR; 1.038,).

**Conclusion:**

The majority of HIV patients with presumptive PTB with smear-negative and Xpert negative results improved on the antibiotic treatment and did not require empiric TB treatment. Initial antibiotic treatment appeared more successful in patients with less advanced HIV disease. Findings from our study suggest it is useful to initiate HIV infected patients with presumptive PTB having smear and Xpert negative results on an initial course of antibiotic treatment prior empiric TB treatment.

## Introduction

Tuberculosis (TB) with HIV co-infection is highly prevalent in sub-Saharan Africa, accounting for seventy-five percent of HIV-associated TB globally [1]. Traditionally, TB diagnosis in the region relies on sputum smear microscopy. However, TB microscopy misses many patients especially if they are infected with HIV [2]. Thus, smear-negative TB is a common clinical problem and the practice of empiric TB treatment is equally common [3, 4]. Since 2010, the World Health Organization (WHO) has recommended Xpert MTB/RIF test (Xpert) as the primary diagnostic for HIV-associated TB or where resources are limited, as a follow-on test to TB microscopy. Xpert has been scaled-up in numerous global settings since that time [5, 6]. However, Xpert has only moderate (67%) sensitivity for smear-negative pulmonary TB and a negative Xpert result is insufficient therefore to rule out active pulmonary TB (PTB) [7, 8]. Existing clinical algorithms for management of ambulatory Xpert negative patients include further investigation for other aetiologies of respiratory disease or extra-pulmonary TB (EPTB) with the assistance of quality chest X-ray, repeat Xpert or culture [9, 10]. These algorithms however, are lengthy to implement, requiring patients to make three or more visits with high potential for patient drop-out during the diagnostic process [11]. Furthermore, quality chest X-ray is not accessible in many peripheral or even referral health facilities. But even when chest X-ray is performed, it does not provide a microbiological diagnosis of TB and cannot therefore, be relied upon for a definitive diagnosis of the disease [12]. On the other hand, Mycobacterial culture is undertaken at central level laboratories and results are seldom available in a clinically relevant timeframe [13]. While a repeat Xpert test although attractive, is currently very expensive to implement in routine patient care [14]. Thus, clinicians in settings where TB-HIV co-infection is prevalent, still face the dilemma of what to do with smear-negative and Xpert negative patients. Commonly, they treat such patients for TB on basis of clinician decision [15, 16]. In the era when TB diagnostic algorithms relied primarily on microscopy, the value of antibiotic treatment in HIV patients with presumptive TB was limited as response to the antibiotics did not exclude TB [17]. While several studies have researched the diagnostic accuracy of the Xpert, there is limited evidence for the use of antibiotic treatment in the diagnosis process of TB in the era of Xpert [18]. In this study, we report on outcomes in our setting in Uganda, following the implementation of an algorithm to manage HIV-infected individuals with presumptive TB who were smear-negative and Xpert-negative. The algorithm involved initial treatment with broad spectrum antibiotics prior a decision to initiate empiric TB treatment. This strategy was previously used before to increase diagnostic accuracy of TB in high HIV prevalent settings [19]. Since the Xpert has moderate sensitivity for smear-negative TB which is common in this patient group, the question of response to initial antibiotic treatment is an important one to study in settings with limited diagnostic microbiology.

## Methods

### Study design and setting

This prospective cohort study was conducted at the Mulago-Immune Suppression Syndrome (Mulago-ISS) clinic which is located at the campus of Mulago National Referral Hospital in Kampala, Uganda. The clinic is a doctor led outpatient HIV care facility supported by the Makerere University Joint AIDS Program (MJAP). Since 2004, the clinic was funded by the U.S. President's Emergency Plan for AIDS Relief (PEPFAR) to provide comprehensive HIV care services including TB, to adults and children at no charge. The HIV care services provided at the clinic include HIV counselling and testing, treatment of HIV related complications, laboratory testing (confirmatory HIV-1 testing, complete blood count and CD4 lymphocyte count), co-trimoxazole prophylaxis, and anti-retro-therapy (ART) according to the World Health Organization and the Uganda Ministry of Health guidelines. Tests for CD4 lymphocyte count were performed routinely for each patient every six months using a FACS Calibur machine (Becton Dickinson, Franklin Lakes, NJ, USA). At the time of the study, tests for viral load monitoring were not performed routinely and were only done in patients suspected of virological failure based on clinical and immunological grounds. As of March 2012, 14,000 patients were receiving HIV care at the clinic. Initiation of ART was done for all HIV infected individuals with a diagnosis of AIDS (HIV clinical stage IV disease) or a CD4 count < 350 cells/mm3 or HIV with TB co-infection or HIV with active - Hepatitis B infection or HIV associated nephropathy. During the study period, an infection control nurse used a Ministry of Health “Intensified TB Case Finding Form” to screen all patients for TB symptoms (cough for two weeks or more, or persistent fevers for two weeks or more, or noticeable weight loss in last one month or excessive night sweats for three weeks or more) as they waited in the seating area

### Patient selection

Participants included consecutive HIV infected patients presenting with presumptive PTB who were smear-negative after fluorescent TB microscopy (FM) between March 2012-June 2013 inclusive of recruitment and final assessment. Participants fulfilled the following criteria: confirmed HIV-1 infection; age ≥ 18 years; cough ≥ 2 weeks or fevers ≥ 2 weeks or noticeable weight loss or excessive night sweats ≥ 3 weeks. Patients who were smear-negative and Xpert negative were enrolled into the study. We excluded patients on quinolone medication during the enrollment period.

### Study procedures

Participants provided a spot and early morning sputum sample which were examined by FM. Samples which were FM negative (smear-negative) were examined further using a one-off Xpert test and combined solid and liquid TB culture as part of a research procedure [20]. If the Xpert test was positive, TB treatment was initiated. Patients whose Xpert test results were negative for TB were treated with oral broad spectrum antibiotics using macrolides such as azithromycin, or cephalosporins such as cefuroxime. We did not use quinolones as they used to treat TB. Patients were asked to return for review after two weeks or earlier if their symptoms worsened. At review, the clinical status of the patients was assessed to establish response (reported by patients as absence or presence of symptoms) to the antibiotic treatment. If the patient did not respond to the antibiotic treatment, other investigations when present, such as chest X-ray or abdominal ultra-sound or microscopy exam of lymph node aspirate or biopsy were done to exclude EPTB and other etiologies of respiratory disease. Patients diagnosed with other respiratory diseases were referred to specialist clinics in the hospital and no further follow-up was done. If no other diagnosis was made, the patients were initiated on standard anti-TB treatment empirically. This comprised an initial phase of two months of daily rifampicin, isoniazid, pyrazinamide and ethambutol (all the Xpert negative patients were presumed to be susceptible to Rifampicin), followed by a four months continuation phase of daily rifampicin and isoniazid at a specialized out-patient TB/HIV clinic. Patients were reviewed after two weeks initially and every 4 weeks subsequently to monitor clinical status and adherence to treatment. All the patients were followed up for six months.

### Study definitions

We defined the baseline CD4 lymphocyte counts as those that were recorded closest to the date when patients were enrolled into the study, with a maximum of 6 months prior. Response to antibiotic treatment was reviewed each month and was defined as being symptom free at six months after the course of antibiotic treatment. Response to empiric TB treatment was defined as being symptom free at six months following initiation of the TB treatment. Duration on ART was defined as the period from the date of initiation of ART at MJAP to the date when the patient was enrolled into the study. Patients were defined as lost to follow-up if they did not attend the clinic for more than 90 days from the day the patient's sputum sample was collected. Individuals recorded as dead in the patient charts were confirmed through telephone call to their next of kin.

### Data collection

A standardized data collection form was used to collect individual patient data at presentation (baseline) and at month six. Socio-demographic data: comprised gender, age, education level, employment status and marital status. Clinical data comprised: baseline CD4 lymphocyte cell, HIV clinical stage, ART treatment status and duration if on ART, history of previous TB treatment and the diagnosis on review at week two. Laboratory data: comprised Xpert test result and TB culture test result. Treatment outcome data comprised: response, no-response, lost-to-follow-up, or dead upon antibiotic or empirical TB treatment.

### Data analysis

The mean and median were used to summarize numerical data. Frequencies and proportions were used to summarize categorical data. The T-test was used to compare normally distributed numerical data and the Mann-Whitney rank-sum test was used to compare non-normally distributed numerical data. The Chi-squared or Fisher's exact tests were used to compare categorical data. We compared the odds of response to antibiotics to the odds of no-response to the antibiotics (inclusive of those diagnosed with other respiratory diseases) to obtain crude odds ratios for each patient characteristic. Multivariate logistic regression using the crude odds ratios was performed to obtain adjusted odds ratios for characteristics with p values of less than 0.2 and those with a biological plausibility to influence response to antibiotic treatment.

### Ethical considerations

The Uganda national Council for Science and Technology provided the ethical approval for the study (HS 1214).

## Results

### General patient characteristics

**Figure 1:**
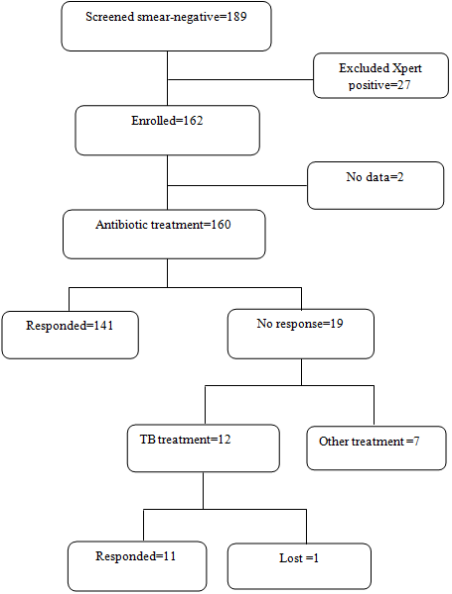
Study flow and patient treatment outcomes

A total of 162 smear-negative and Xpert-negative patients were enrolled. Their general characteristics are summarized in Table 1. Of the cohort, 59% (96 of 162) were female. The mean age was 38 years (SD ± 10), the median CD4 lymphocyte count was 293 cells/µL (IQR: 129-455), and 81% (131 of 162) were on ART at enrolment. The median duration on ART was 8.7 months (IQR: 2.9-30.3). A small majority, 56% (90 of 162) were in late HIV clinical stage (III and IV). Other socio-demographic characteristics are presented in the table. Of the 162 smears and Xpert negative patients, 99% (160 of 162) received broad spectrum antibiotics while treatment data was unavailable for two (Figure 1). Among these, 88% (141 of 160) responded to the treatment. Conversely, 12% (19 of 160) did not respond the treatment. Of the 19 who did not respond to the antibiotic treatment, seven were diagnosed with other respiratory illnesses (Kaposi sarcoma-one, bronchial-alveolar carcinoma-one and chronic obstructive pulmonary diseases-five) while twelve got empiric TB treatment to which the majority (11 of 12) responded. As shown in Table 2, the odds of improvement on antibiotics were not significant with any of the socio-demographic or clinical variables tested except for stage of HIV. The odds were lower (0.05) and statistically significant in patients with advanced HIV disease than in patients with early HIV disease (OR 0.05; 95% CI 0.0-0.42). The significance was retained in a multivariate model (Table 3). Of the cultures requested, reports were provided for 68% (110/162) of the patients. Nearly all the missing reports were for patients who received antibiotic treatment only. The culture reports were negative for TB in all patients who responded to antibiotic treatment and in patients diagnosed with other respiratory illnesses. Of the twelve patients who got empiric TB treatment, only one was culture positive, eight were culture negative and three were undetermined because the cultures were either contaminated or the report was missing.

**Table 1:** General patient characteristics

Characteristic	Counts (N = 162)	Percent (n/N)
Age: years, mean, (SD)	38 (10)	-
Males	66	41
Females	96	59
CD4 cell count/µL, median (IQR)	293(129-455)	-
HIV Early Clinical Stage (1&2)	72	44
HIV Late Clinical Stage (3&4)	90	56
On ART	131	81
Not on ART	31	19
Duration on ART: months, median, (IQR)	8.7 (2.9-30.3)	- -
Had previous TB treatment	24	15
No previous TB treatment	138	85
Primary education level and below	102	63
Secondary education level and above	60	37
Not employed	20	12
Employed	142	88
Never married	13	8
Married	88	54
Separated/widowed	61	38

**Table 2:** Patient characteristics compared by response to antibiotics

Characteristic	Improved on antibiotics (N = 141)	No improvement on antibiotics (N = 19)	Odds ratio (95% CI)	p-value
Age: years, mean, (SD)	38, (9.9)	38, (11)	0.99 (0.95-1)	0.991
Males, no. (%)	57, (40)	7, (37)	0.86 (0.31-2.3)	0.765
Females no. (%)	84, (60)	12, (63)		
CD4 count ≤350 cells/µL, n (%)	83, (59)	13, (68)	1.5 (0.5-4.2)	0.427
CD4 count >350 cells/µL, n (%)	58 (41)	6, (32)		
HIV Early Clinical Stage (1&2) n, (%)	71, (50)	1, (5)	0.05 (0.0-0.42)	0.005
HIV Late Clinical Stage (3&4) n, (%)	70, (50)	18, (95)		
On ART, n, (%)	112, (79)	17, (89)	2.2 (0.48-10)	0.309
Not on ART, n (%)	29, (21)	2, (11)		
Duration on ART: months, median, (IQR)	14, (6.1- 1.2)	7.8, (4.6 – 10.4)	1 (0.99-1.06)	0.096
Had previous TB treatment, no, (%)	21, (15)	3, (16)	1.1 (0.29-4)	0.918
No previous TB treatment, no, (%)	120, (85)	16, (84)		
Primary education and below, no, (%)	88 (62)	12 (63)	1 (0.38-2.78)	0.950
Secondary education and above, no, (%)	53 (38)	7 (37)		
Not employed, no, (%)	16 (11)	4 (21)	2.1 (0.62-7.05)	0.238
Employed, no, (%)	125 (89)	15 (79)		
Never married, no, (%)	12 (9)	1 (5)	1	-
Married no, (%)	79 (56)	8 (42)	0.82 (0.09-7.18)	0.860
Separated/widowed no, (%)	50 (35)	10 (53)	0.42 (0.49-3.58)	0.425

**Table 3:** Adjusted odds ratios of patient characteristics for response to antibiotics

Parameter	Crude odds ratio (95% CI)	p-value	Adjusted odds ratio (95% CI)	p-value
Age	0.99 (0.95-1)	0.991	0.978 (0.930 - 1.028)	0.381
Gender	0.86 (0.31-2.3)	0.765	0.760 (0.251 - 2.299)	0.627
CD4 cell count	1.5 (0.5-4.2)	0.427	0.668 (0.171 - 2.618)	0.563
HIV clinical stage	0.05 (0.0-0.42)	0.005	0.038 (0.005 - 0.307)	0.002
ART status	2.2 (0.48-10)	0.309	5.016 (0.699 - 36.001)	0.109
Duration on ART	1 (0.99-1.06)	0.096	1.038 (1.003 - 1.074)	0.032
Previous TB treatment	1.1 (0.29-4)	0.918	0.577 (0.139 - 2.393)	0.448

## Discussion

We studied outcomes following the implementation of a simple algorithm to manage smear-negative and Xpert-negative HIV patients with presumptive PTB in a routine HIV clinical setting with limited TB diagnostics. This involved treatment for bacterial infection, clinical assessment if there was no response, and followed by empiric TB treatment. The majority (88%) of the patients responded to treatment for bacterial infection with only a few (8%) getting empiric TB treatment. Our study suggests, that a negative smear and Xpert had a very high negative predictive value for PTB in our clinic (only one culture positive case out of 110). Treatment with broad spectrum antibiotics could therefore be useful to exclude TB in HIV patients in our setting. In our study, treatment for bacterial infection appeared to be useful in individuals with CD4 cell count > 350, or in early stage HIV disease (and therefore not eligible for ART) although some of this lost significance in multivariate analysis. Conversely, in individuals with CD4 cell count ≤ 350, or in late stage HIV disease (and therefore eligible for ART), undiagnosed TB remained a possibility despite a negative smear and Xpert result and such patients benefited from the empiric TB treatment. When we compared the clinicians’ decisions to initiate empiric TB treatment with TB culture results, only one of twelve patients had culture confirmed disease. The decision to initiate TB treatment in these patients therefore poorly correlated with the culture results as reported previously [3]. However this level of empiric TB treatment was comparable to that in a study from South Africa where 11% of HIV patients with presumptive TB and Xpert negative results also got treated for TB empirically [21]. It is likely that the patients in our study whose cultures were negative had very few TB bacilli in their clinical samples. The viability of these bacilli could have been reduced or some even killed by the procedures involved in processing the samples for TB culture [22]. This possibility was supported by a sub analysis of the data from our diagnostic accuracy study of Xpert which revealed nearly half of samples with very low MTB detected results, had cycle threshold values of thirty or more and negative TB culture results [20]. While there are concerns about the use of antibiotic treatment as a diagnostic aid because of treatment delay and antibiotic resistance particularly for TB [23], the benefits of antibiotic treatment could outweigh the risks because of reduced empiric TB treatment and the associated adverse consequences of the practice such as; drug toxicity, long treatment duration and increased possibility of generating multi drug-resistant TB. Moreover, patients are more likely to complete short-course antibiotics regimens as prescribed, which is one strategy to decrease antibiotic resistance [24]. In addition, the effectiveness of the antibiotics could be maintained if they are chosen wisely. For example, in our study, we excluded the use of quinolones because they are used in second line treatment for TB, and their routine use is associated with delayed treatment and resistance in TB [25]. To facilitate wise choice of the antibiotics, clinicians need to follow existing recommendations to prescribe fewer broad spectrum antibiotics based on standard treatment guidelines. These guidelines in turn need periodic and regular review using data from surveillance of bacterial resistance and antibiotic use [26].

Our strategy could be useful in our setting or similar other settings because laboratories may not cope with the influx of clinical specimens for both TB diagnosis and follow-up examinations. This strategy could therefore save resources, that would otherwise be consumed by performing additional tests and procedures for patients in whom the majority would eventually not have TB as shown by one study from the same study area [27]. This study and another recent one, showed that the common bacterial infections in HIV patients, are susceptible to broad spectrum antibiotics and therefore are useful in routine patient care [28]. Moreover, it was shown also, that the early clinical consequences of false-negative Xpert tests were not grave and that rapid empiric TB treatment may not be necessary in patients with Xpert negative results [21]. Our strategy involved two clinic visits by patients before the decision to treat for TB was taken. Few patients (3 out of 162) were not accounted for in the diagnostic pathway meaning the strategy could be feasible. This is useful because lengthy algorithms are poorly implemented by clinicians and are associated with high patient loss-to-follow-up and poor patient outcomes [11, 29]. Our study was done in a setting where Xpert had a high negative predictive value (those who were negative by Xpert were unlikely to have PTB) and therefore, cure from a course of antibiotics was high. The results are only applicable to similar settings where Xpert has a high negative predictive value. We acknowledge the small size of our study, which could result in inconsistent measures of association. Second, we studied a highly selected out-patient HIV population where the majority (81%) of the individuals were on ART compared to 62% in the general adult population in Uganda setting [30]. These results may therefore not be generalized to hospitalized patients or to populations where ART coverage is low. Third, our findings could also be cofounded by co-morbidities such as smoking status, diabetes, adherence to the prescribed medicines or baseline CD4 versus CD4 counts at six months, which we did not analyse as these data were not collected. Other predictive factors not collected included symptom intensity, haemoglobin concentration, serum C-reactive protein and body mass index, Therefore, the range of possible factors influencing patient outcomes in our study was not exhaustive. Fourth, we did not ascertain if the patients whose cultures were contaminated and were empirically treated for TB, did not actually have disease. Additional sample collection and culture would have been useful to confirm if they had TB or not. However, the results were not received in time to collect another sample before treatment was initiated.

## Conclusion

The majority of HIV patients with presumptive PTB with smear-negative and Xpert negative results in our clinic setting improved on the antibiotic treatment and did not require empiric TB treatment. Initial antibiotic treatment appeared more successful in patients with less advanced HIV disease. Findings from our setting suggest it is useful to initiate HIV infected patients presenting with presumptive smear negative PTB on an initial course of antibiotic treatment.

### What is known about this topic

- The Xpert MTB/RIF test (Xpert) detects about 80% of pulmonary TB cases in people living with HIV (PLHIV).
- Xpert also improves the quality and turnaround time for TB diagnosis among PLHIV who are smear microscopy negative.
- However, when PLHIV are both smear and Xpert negative, the role of empiric TB treatment is unclear in settings where additional investigations for TB are limited.

### What this study adds

- In this study, we show that routine practice of empiric TB treatment, in PLHIV who are both smear and Xpert negative is un-necessary.
- Majority of such patients respond fully to broad spectrum antibiotic treatment that covers typical and atypical bacteria.
- Empiric TB treatment only appears necessary when targeted at individuals in HIV clinical stage 3 and 4, after close follow-up.
